# The Healthy Fatty Index Allows for Deeper Insights into the Lipid Composition of Foods of Animal Origin When Compared with the Atherogenic and Thrombogenicity Indexes

**DOI:** 10.3390/foods13101568

**Published:** 2024-05-17

**Authors:** Alessandro Dal Bosco, Massimiliano Cavallo, Laura Menchetti, Elisa Angelucci, Alice Cartoni Mancinelli, Gaetano Vaudo, Stefania Marconi, Emanuela Camilli, Francesco Galli, Cesare Castellini, Simona Mattioli

**Affiliations:** 1 Department of Agricultural, Environmental and Food Science, University of Perugia, Borgo XX Giugno 74, 06124 Perugia, Italy; alessandro.dalbosco@unipg.it (A.D.B.); elisa.angelucci@unipg.it (E.A.); alice.cartonimancinelli@unipg.it (A.C.M.);; 2 Department of Medicine and Surgery, University of Perugia, Piazzale Gambuli 1, 06132 Perugia, Italy; massimilianocavallotr@gmail.com (M.C.); gaetano.vaudo@unipg.it (G.V.); 3 School of Bioscience and Veterinary Medicine, University of Camerino, Via Circonvallazione 93/95, 62024 Matelica, Italy; laura.menchetti@unicam.it; 4 Council for Agricultural Research and Economics, Research Centre for Food and Nutrition, 00178 Rome, Italy; stefania.marconi@crea.gov.it (S.M.); emanuela.camilli@crea.gov.it (E.C.); 5 Department of Pharmaceutical Sciences, University of Perugia, 06126 Perugia, Italy

**Keywords:** fatty acids, indexing, healthy quality, animal products

## Abstract

The aim of this research was to validate the effectiveness of the Healthy Fatty Index (HFI) regarding some foods of animal origin (meat, processed, fish, milk products, and eggs) typical of the Western diet and to compare these results with two consolidated indices (atherogenic—AI, and thrombogenic—TI) in the characterization of the nutritional features of their lipids. The fatty acids profile (% of total fatty acids and mg/100 g) of 60 foods, grouped in six subclasses, was used. The AI, TI, and HFI indexes were calculated, and the intraclass correlation coefficients and the degree of agreement were evaluated using different statistical approaches. The results demonstrated that HFI, with respect to AI and TI, seems better able to consider the complexity of the fatty acid profile and the different fat contents. HFI and AI are the two most diverse indices, and they can provide different food classifications. AI and IT exhibit only a fair agreement in regards to food classification, confirming that such indexes are always to be considered indissolubly and never separately, in contrast to the HFI, which can stand alone.

## 1. Introduction

Dietary lipids are important sources of energy and other bioactive substances for humans and animals, providing fatty acids (FA) of different natures and metabolic functions, depending on the presence, number, and position of double bonds [1]. However, over time high-fat foods have been strongly demonized because great attention was paid to fat quantity rather than quality [2,3]. In particular, polyunsaturated fatty acids (PUFA), particularly those of the n-3 series, have positive health effects for reducing the risk of several diseases, such as coronary heart disease and strokes, chronic inflammation, and several cancers, and are important for the development and functionality of the brain and retina, along with reproductive functions [4,5].

In addition, for human nutrition, it is important to consider not only the fatty acid composition of foods, but also the relationships between the various PUFA classes [6,7,8]. Indeed, current Western diets result in an excessive intake of n-6 PUFA and a high n-6/n-3 ratio, which can promote several diseases, such as diabetes, depression, immune disorders, and neurological dysfunctions [9], whereas, the role of the unsaturated fatty acids (UFA) in inhibiting plaque accumulation and reducing blood cholesterol levels has been well documented [10].

In such a complex scenario, researchers and food producers are increasingly focused on the production of healthy foods. Many approaches have been developed for determining how to classify the nutritional properties of lipids in different foods. Recently, the Nutri-Score has been introduced in some EU Countries [11], a nutrition label using graded color-coding to provide a simple nutritional food score. The Nutri-Score demonstrates high discriminating ability for all groups of food, with similar trends, e.g., fruit and vegetable were mainly classified in the two healthiest categories, while sugar and animal fats were mainly classified in the two less-healthy categories [12]. However, for many high fat products, this classification may be incorrect and misleading; therefore, the Nutri-Score has not been considered reliable in some countries [13].

From this point of view, the relationship between the FA composition of food and its nutritional and health value should be better evaluated. In the belief that the determination of the fatty acid profile, especially if expressed as a percentage, is not sufficient to explain the nutritional value of foods, in a previous review article [14] we traced the state of the art approach in regards to the nutritional lipid indexing of poultry meat. The approach of this new lipid index is that the healthiness of food does not depend on how many FAs deemed beneficial (n-3 PUFA) it contains, but on a much more complex situation, involving the consideration of lipid content and relationships between SFA, MUFA, and different classes of PUFA.

Therefore, the aim of this research was to verify the effectiveness of this healthy fatty index (HFI) in some foods of animal origin (meat, processed, fish products, milk, eggs, etc.) representative of the Western diet and to compare these results with the application of two consolidated indices (atherogenic and thrombogenic potential).

## 2. Materials and Methods

### 2.1. The Dataset

The CREA (Food and Nutrition Research Center) dataset (https://www.alimentinutrizione.it/tabelle-nutrizionali/ricerca-per-alimento, accessed on 1 April 2023) was used. This dataset includes most of the data on the composition of products obtained over the last ten years and represents a total review of published and validated data. Only a small portion of data was derived from an accurate bibliographic selection, mainly Italian, and about 2% of the data is calculated or estimated starting from similar foods. In addition, in this trial, data on rabbit and poultry breast meat of two genetic lines (slow-growing and fast-growing) were taken from our own previously papers [12]; the same is true for data regarding eggs enriched with n-3 and rabbit [15,16].

Although the values are derived from analyses of representative samples of food, it should be underlined that the food composition presents a natural variation, and it is subject to several factors regarding differences. Season, type of feed, rearing system, and storage conditions are among the main factors that can influence the composition of animal foods, just as a difference in water content in 100 g of the same food strongly affects the concentration of all the nutrients. The samplings methods are not identical for all the studied foods, and scientific caution has been taken to obtain comparable results.

The data is also expressed for 100 g of the edible portion (percentage of the food that is consumed after removing the waste) and for one portion. The portion was quantified in compliance with Italian food tradition and the expectations of the consumer, considering the recommendations of the intake level of the various nutrients [17] and the guidelines for a healthy diet [18]. For some foods where is impossible or wrong to quantify a portion, the data are reported per 100 g. For the calculation of the energy (kcal), the recommendations of Greenfield and Southgate [19] were followed.

The study was exclusively focused on the lipid amount and fatty acid profile of the considered foods, although on some occasions, marginal and non-indexical aspects of the recommended dose were considered.

A total of 60 representative foods of animal origin were selected, according to the following categories: fresh meat, processed meat, fish/shellfish/crustaceans, cheeses, and eggs. The fatty acids of these foods (expressed as a % of total fatty acids) were taken from the dataset, while the mg of fatty acids/100 g of food were obtained with the conversion factors suggested by Weihrauch et al. [20] (Tables S4–S16).

### 2.2. The Comparison of Indexes

#### 2.2.1. Atherogenicity Index (AI)

AI = [C12:0 + (4 × C14:0) + C16:0]/(UFA)(1)

This index was developed by Ulbricht and Southgate in 1991 [21], with the aim to characterize the atherogenic potential of foods. The two researchers wanted to define a more complex index compared to the PUFA/SFA, which was considered too general and only a weak indicator of the atherogenicity of food [22]. The AI has been widely used for evaluating seaweeds, crops, meat, fish, and dairy products.

#### 2.2.2. Thrombogenicity Index (TI)

TI = (C14:0 + C16:0 + C18:0)/[(0.5 × MUFA) + (0.5 × n-6) n-3) + (n-3/n-6)](2)

This index was also developed by Ulbricht and Southgate [21], together with AI, to further characterize the thrombogenic potential of FAs, separating them based on the effects triggered by some derivatives (eicosanoids) in pro-thrombogenic (C12:0, C14:0, and C16:0) and anti-thrombogenic FAs, such as MUFA, n-3, and n-6 PUFA [23].

#### 2.2.3. Healthy Fatty Index (HFI)


(3) $$HFI=\frac{(MUFA\times 2)+(n-6\times 4)+(n-3\times 8)+\frac{n-3}{n-6}}{(SFA\times 1)+(MUFA\times 0.5)+(n-6\times 0.25)+(n-3\times 0.125)+\frac{n-6}{n-3}}$$

All the values were expressed in mg/100 g.

In this index, we differentiated the various classes of FA (by unsaturation and by the position of the double bonds), partly following the indications of Ulbricht and Southgate [21] but also considering the different classes of FA and their role in cardiovascular diseases (CVD). The rationale of this index is to underline recent knowledge on the nutritional and health value of some fatty acid classes in regards to CVD onset, not only based on their biological and metabolic properties, but also considering their quantities weighted with multiplication coefficients. In the numerator are reported the FA classes multiplied by the relative positivity coefficients, while in the denominator, the FA classes are multiplied by fractional coefficients of negativity, except for the SFA, which are multiplied by 1, to indicate the maximum level of attention for the aforementioned diseases.

The HFI, in contrast to the AI and TI, is a direct index because the higher values correspond to healthier foods.

### 2.3. Statistical Analyses

Descriptive statistics were used to present the data. Then, the data were standardized (by subtracting the mean of the measure and dividing by its standard deviation) [24], and the AI and TI were multiplied by −1 (because they have an opposite interpretation compared to the HFI). Each food index was also categorized, using tertiles, into three levels by applying the statistical binning procedures of SPSS software (i.e., Low HFI/Low IA/Low IT, Medium HFI/Medium IA/Medium IT, High HFI/High IA/High IT; outliers were included; Tables S1–S3; [25]). Binning created a new, categorical variable for each index by splitting their values. The binning criterion was based on percentiles and, as there were three categories (i.e., bins), each bin included 33.3% of the evaluated foods. Therefore, analyses of agreement were carried out both on the standardized values of the indices (continuous variables) and on the categories created with binning (categorical variables).

Intraclass correlation coefficients (ICC) were calculated to assess the agreement between the different indices evaluated as continuous variables. ICC used the two-way ANOVA approach, and values for single measurement and absolute agreement type were reported. Its values were interpreted as poor (ICC < 0.40), fair (0.40 ≤ ICC < 0.60), good (0.60 ≤ ICC < 0.75), and excellent (ICC ≥ 0.75) [26]. The agreement between index classes (i.e., categorical variables—Low, Medium, and High) was evaluated by McNemar’s test and Cohen’s kappa. Agreement was poor if k < 0.00, slight if 0.00 ≤ k ≤ 0.20, fair if 0.21 ≤ k ≤ 0.40, moderate if 0.41 ≤ k ≤ 0.60, substantial if 0.61 ≤ k ≤ 0.80, and almost perfect if k > 0.80 [27]. The multivariable polynomial regression curve between HFI and AI or TI was also reported (95% upper and lower limits).

To evaluate the predictive strength of the different indices and the relative importance of their predictors (individual fatty acids), the Automatic Linear Modeling (ALM) method was used. After checking for multicollinearity with VIf and tolerance statistics, the percentage of SFA and n-3 were included in the model as predictors. The ALM finds the best predictive model using the available data, provides information on the accuracy of the model (equivalent to the adjusted R-squared value and information criterion (AIC)), and the importance of each predictor (residual sum of squares with the predictor removed from the model and normalized).

Data were analyzed using SPSS software version 25 (SPSS Inc., Chicago, IL, USA), and *p* values < 0.05 were considered statistically significant.

## 3. Results and Discussion

Fat is an important nutrient in the diet, providing energy, bioactive compounds, and palatability to foods, or serving as a cooking medium [28]. However, some foods rich in fat have differing health impacts, mainly due to the relationship between SFA, MUFA, and PUFA [3,29].

Fatty meats are generally considered foods with low-fat quality [30], whereas fish products generally have good fat quality [31,32]. However, this is a crude classification which requires further details from the point of view of lipid characteristics. This study aimed to explore the nutritional impact of some animal-origin products, including how/whether it is possible to design them as more or less healthy.

First of all, the general trend of indexes and the agreement between them have been analysed.

### 3.1. Analyses of HFI, AI, and TI Agreement

The ICC values indicated a fair agreement between HFI and AI, whereas the agreement was excellent between TI and AI, as well as between HFI and TI (Table 1; *p* < 0.001). These values dropped when foods were stratified according to HFI classes. In these latter cases, the agreement was always poor between HFI and AI, and for all indices, when the HFI value was medium and high. The stratification of the dataset in regards to HFI classes reduced the sample size for each analysis, even if the ICC was not particularly sensitive to sample size. Thus, the low ICC cannot be explained only by the sample size, but it also highlights the disagreement between some indices and for each index, the influence of the magnitude of its values. In particular, these findings confirm the different approaches/meanings of HFI and AI. In fact, the AI is concentrated on the atherogenic characteristics of some fatty acids (e.g., myristic acid is multiplied by four), and the residual fatty acid composition of the food, a part of a generic UFA, is completely ignored. The situation is somewhat different for TI, where the classes of fatty acids are separated in the n-3 and n-6 PUFA series.

Then, the agreement was also estimated for the categorical variables created by binning using the McNemar–Bowker’s test and Cohen’s kappa (Table 2 and Tables S4–S6). The aim was to evaluate whether, regardless of the agreement of the values, the three indices are able to provide similar broad indications regarding the healthiness of foods classified into three different groups (i.e., Low, Medium, and High HFI).

The non-significant result of the McNemar–Bowker test suggested that there is no evidence of a systematic difference between the indices, but the Cohen’s kappa test confirmed a fair agreement between the classification of foods established by HFI and AI. Again, the agreement was nearly confirmed between HFI and TI. These findings underline that HFI and AI contain different food info and that, unexpectedly, they can provide contrasting indications regarding the classification of foods. However, it is interesting to unnote that even AI and TI only have a fair agreement, confirming that the two “historical” indexes of Ulbricht and Southgate should be considered together, contrary to the HFI, which is a single index for evaluating foods.

**Figure 1:**
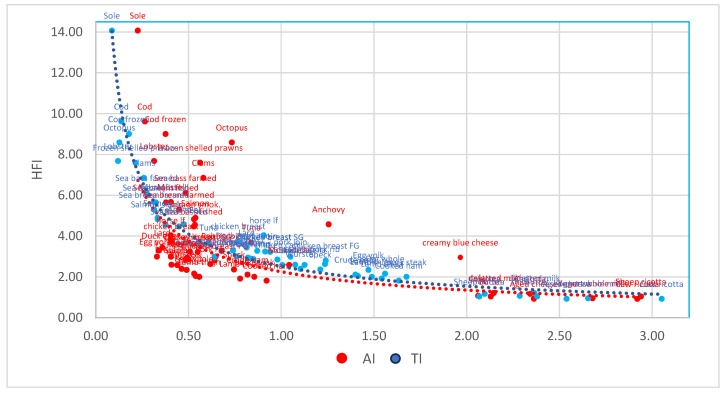
Multivariable polynomial regression curve between HFI and AI (in red) or TI (in blue) (95% upper and lower limits).

In Figure 1, a more direct assessment of the relative trends of the relationship between the HFI-AI-TI indexes is shown. In particular, as previously mentioned, the indexes show a general agreement between them: high HFI values correspond to low AI and TI levels and vice versa, even if—for HFI-AI—the distribution is less close, with several outliers (i.e., creamy blue cheese, octopus, anchovy).

Generally speaking, foods at the extremity of the curve (e.g., high HFI: fish; low HFI: milk and milk products) show a good consonance, while meat and meat products show a lower agreement. Notably, AI tends to underestimate the nutritional and health values of meat and meat products (judged as less healthy, whereas the opposite is true for TI.

The trend is affected by the different dependency of the indexes on the lipid content and FA profile of foods. The major discrepancies apply to foods with uncommon/extreme FA profiles (e.g., very high or very low proportion of SFA or n-3 PUFA—see Figure 1), which play a different role in the formation of the indexes.

The automatic linear modeling approach confirmed that HFI is the best model to account for the SFA and n-3 in the food (accuracy **=** 87.3%, 86.0%, and 78.4% for HFI, IA, and IT, respectively). Furthermore, the importance attributed to SFA and n-3 varies according to the index. In fact, while the most important predictor for HFI is the n-3 value (87%), the percentage of SFA has a greater weight for the other two indices (importance of n-3: 15% and for IT and IA, 5%, respectively; Table S7).

This trend may be interesting to discriminate between enriched (n-3 enriched egg, n-3 enriched milk [33], etc.) [32] or uncommon (very extreme food, according to the FA profile: e.g., suet rich in SFA) foods, because in these foods, the HFI index showed a better discriminating power, than that of either AI or TI.

After a general discussion regarding the tendency and agreement between the indexes, we briefly analyze the trend within each food category. For each food category, we underline the best (green text) or worst (red text) foods for the three indexes by evaluating which class (or fatty acid) has the most significant impact.

### 3.2. Fresh Meat

Concerning fresh meat, fifteen different commercial cuts belonging to various species of livestock diffused in Western countries were considered (Table 3). The best and the worst meat within each index have been highlighted.

The consumption of a certain quantity of meat, and in particular red meat, is commonly defined as having a negative effect on human health [34,35]. However, specific literature shows a high degree of heterogeneity and uncertainty concerning the effect of red meat, finding a weak association between the consumption of unprocessed red meat and human health [36]. The authors noted that the available data do not permit the confirmation of a consistent association between the consumption of unprocessed red meat and ischemic and hemorrhagic stroke.

As specified above, a sound index of the fatty acid profile of meat could be a valid tool for better understanding the relationships between the characteristics of a meat and the aforementioned cardiovascular diseases. For these products, the comparison of the three indices exposed a clear difference between the different cuts/species. In particular, the best indexes (green marks and green highlights) mainly depends on the cut of meat, and it is less dependent on the animal species. Low-fat horse meat has the best HFI (4.04), while lamb chop (1.92) has the worst. As for AI and IT, the best results were associated with pork loin and, unexpectedly, horse meat with fat (0.40 and 0.76, respectively), and the worst results were obtained for the pork steak (0.85 and 1.68, respectively). The reasons for this difference between the various indices, as previously mentioned, originate from the approaches of the indexes.

First of all, it must be emphasized that the HFI considers not only the FA profile, but also takes into account the lipid content of the food, whereas in the AI and TI, only the FA profile is evaluated. In fact, the low-fat horse meat is, along with rabbit loin, the leanest meat of the panel sample. However, if we consider meats with the worst HFI (i.e., lamb chop), it can be seen that this is not the fattest meat of the panel (2.7 g/100 g meat, Table 3). It is therefore obvious that the fatty acid composition has a great impact on the index, and therefore, lean meat is certainly of good nutritional quality, but this is not always the case.

The opposite HFI score between lamb chop and horse meat is mainly due to enormous differences in MUFA and PUFA (n-3 included): 39.56 vs. 15.19 for MUFA, 13.89 vs. 50.63 for PUFA, and 0.42 vs. 3.51 for n-3, respectively (Table S8). Even with a nearly one-third lower lipid weight, the weight of PUFA (Table S9) was strongly influential in the case of low-fat horse meat. As previously shown, HFI “rewards” the low SFA and the high levels of PUFA, obviously including the n-3 values, proving to be an index that enhances these nutritional pillars.

Moving on to the discussion of the other two indices, the best AI was obtained (Table 3) for pork loin, which has a low content of myristic (multiplied by four) and stearic acid; moreover, neither is the palmitic particularly high. This meat possesses 60% of UFA (Table S8), computed in the denominator of HFI index, which further lowered the index. On the contrary, the worst value of AI found in pork steak was due to the high content of myristic acid and the low level of UFA (about 52%; Table S8). Chen and Liu [37] reported detailed information on the AI in many foods, i.e., the AI of seaweeds ranges from 0.03 to 3.58, from 0.08 to 0.55 for crops, from 0.21 to 1.41 for fish, and from 0.17 to 1.32 for meat.

Unexpectedly, the best IT value was observed in horse meat with fat (6.8 g of fat per 100 g of meat; Table 1). This meat has a high amount of MUFA (10.34% of palmitoleic acid and 34.26% of oleic acid; Table S8) and high level of n-3 PUFA (5.01% of linolenic acid), which contributed to lowering the value. The worst IT value confirmed what had been observed for AI, i.e., that pork steak expressed the worst values, which, as mentioned above, is characterized by high level of SFA, which greatly increases the numerator in a way that cannot be reduced by the positive effect of MUFA, n-3, and n-6 PUFA.

As a general consideration regarding fresh meats, we can state that the AI and TI indexes are concentrated on specific possible risks for human health, not fully considering the complexity of the whole fatty acid profile, nor the fat content.

This, with the same recommended dose, seems to represent a critical point in the general application of these indicators. Accordingly, the HFI produces a more complete view of the nutritional potential of a meat by including all the classes of FA and by considering the amount of fat in which they are concentrated or diluted [3].

### 3.3. Processed Meat

Nine processed meat products deriving from red or pork meat transformed through salting, curing, smoking, or other processes were chosen (Table 4). The effect of processed meat on human health [38] has been analyzed in numerous studies, and many authors reported that processed meat, with the aim of improved preservation and enhanced flavor, are subjected to additives, such as sodium, nitrites, nitrates, and phosphates, which could have detrimental effects on human health. Also, Soladoye et al. [39] add new reflections on how the above-mentioned processes can affect the physicochemical properties of the meat, compromise its nutritional components, or produce some compounds of health concern. In particular, protein and lipid oxidation potentially produce reaction compounds which are particularly harmful to human health and, according to the index of fatty acids (the subject of this manuscript), a worsening of their profile and in particular, of the PUFA.

In this specific case, the application of the indices triggers interesting reflections; in particular, the most striking and surprising result is certainly the best scores obtained by pork lard.

The fatty acid profile of lard is very balanced, with a practically equal division between SFA, MUFA, and PUFA (33.45, 37.52, and 29.06 respectively; Table S10); moreover, the PUFA has high contents of linoleic acid and linolenic acid (22.35 and 2.54 g/100 g of edible product). We must remember that, as previously mentioned, the resulting data should be integrated, according to the calories and the recommended daily dose. As an example only (since they are not items indexed in this study), we can say that, compared to other kinds of processed meat, lard has a much lower recommended daily dose than other products (10 g) by virtue of the very high lipid content; however, by analyzing its fatty acid profile, it can be deduced that it is very balanced, with a practically equal division between SFA, MUFA, and PUFA (33.45, 37.52, and 29.06 respectively).

Obviously, the results for bresaola, which is a very lean cut of meat (2% of lipids), are not surprising, while the good values obtained for salami and pure pork wurstel are a bit surprising, compared to those of ham and speck, even the very thin cooked ham registered a complete assonance between the three indexes, positioning itself in the last place of the ranking.

From an atherogenic point of view, because the salami and pure pork wurstel are characterized by a fatty acid composition strongly oriented towards monounsaturated fats, with values of oleic acid alone much higher than 40% of the total FA (Table S10), this enormous incidence affects the entire thrombogenic index.

As previously mentioned, the worst results for all three indices were observed for cooked ham; although this food has an average lipid content for foods within the category (11.8 g/100 g of food; Table 4), it has a very high impact for SFA, with 22.60% of palmitic acid, and a very low level of PUFA (lower than 10%; Table S11).

### 3.4. Fish/Shellfish/Crustaceans

Seventeen fish, shellfish, and crustaceans were considered and compared (Table 5). The consumption of fish is suggested to have a positive effect on human health [40], and this is generally confirmed by all the indices, as previously shown in Figure 1 (localization of higher HFI and lower AI and TI values).

However, the comparison of different foods led to some interesting results. The first differences are the recommended daily doses, which are higher than those for the previous foods (fresh and processed meat), as well as the great variability in terms of lipid content between the various fish, as well as, in general, the high HFI values and the low AI and TI values.

The HFI values were higher than those of the previous foods analyzed, but also in this case, large differences were noted between the different fish foods, with a good correspondence among the three indices. In particular, sole recorded the best HFI, as well as the best AI and IT (0.23 and 0.09, respectively) results.

The sole is a very lean fish (1.40), which has an excellent fatty acid composition with a PUFA level of 64.12%, of which 49.51 is made up of n-3 (Table S12). Moreover, the low content of myristic, palmitic, and stearic acids (29.56, 116.67, and 29.56 mg/100 g, respectively, Table S13) explains the good score obtained.

Tuna and anchovies, two oily fishes, showed the worst values and in particular, tuna for HFI and TI, and anchovy for AI. Analyzing their acid profiles, the reason for this result is clear: they are the fish with the highest SFA content.

### 3.5. Milk and Milk Products

Nine milk and dairy products were chosen (Table 6; Tables S14 and S15). These foods have long been considered as being excellent sources of nutrients in Western countries. Tunick and Van Hekken [41] reported that the benefits go far beyond the improvement of bone metabolism, such as previously unknown benefits for gastrointestinal health and the immune system. Thorning et al. [42], considering the increase in degenerative diseases linked to bad eating habits over recent decades, reviewed the latest evidence from meta-analyses and observational studies regarding the intake of dairy products and the risk of obesity, type 2 diabetes, cardiovascular disease, osteoporosis, and cancer, as well as all-cause mortality. The authors showed that the intake of dairy products can contribute to meeting nutrient recommendations and may protect against the most prevalent, chronic, non-communicable diseases, at the same time highlighting few adverse health effects.

Despite these general reassurances, the lipid indices of milk and dairy products are higher than those of the previously considered foods.

### 3.6. Eggs

As Réhault-Godbert et al. [43] stated, egg is an encapsulated source of macro and micronutrients, meeting all the requirements to support embryonic development until hatching, but also reflecting a perfect balance between nutrients, high digestibility, and affordable price, thus indicating it a basic food for humans. However, eggs are still the subject of significant attention from nutritionists, often aimed at restricting their consumption to limit the incidence of cardiovascular diseases.

Recent literature (epidemiological data, meta-analysis, and clinical interventions) confirmed that there is not a direct correlation between the dietary intake of cholesterol and blood cholesterol due to the activation of several compensatory mechanisms exerted by the body [44]. Most of the studies indicate that dietary cholesterol is not associated with CVD risk nor with high plasma cholesterol concentrations. Further, when eggs are the source of dietary cholesterol, the formation of fewer atherogenic lipoproteins and a more efficient cholesterol transport is observed. However, if the cholesterol sources are associated in diet with high SFA and trans fats increases in plasma cholesterol may be observed.

The egg remains a food of great nutritional quality for humans, and it is widely consumed throughout the world. There is even scientific evidence demonstrating that the egg also contains many bioactive compounds [16,45] that could be of great interest in the prevention/treatment of diseases. In this study, we compared four products (Table 7). The results highlight that laying hen and duck eggs differ considerably, both in lipid content and fatty acid profile; although the egg of the duck is fattier than that of the hen, the lower level of SFA and the higher level of MUFA and PUFA (in particular LA and ALA) have resulted in the best HFI, AI, and TI values (Table 7, Tables S16 and S17).

## 4. Conclusions

There is a large body of literature regarding the use of the AI and TI to estimate the human risk of CVD correlated with the fat characteristics. The AI mainly takes into consideration FA, with the anti-atherogenic activity (PUFA), as these inhibit the accumulation of cholesterol plaque and reduce the phospholipids, blood cholesterol, and esterified FA [22]. In this regard, our results confirm that the pork loin, bresaola, sole, gorgonzola, and duck egg may be considered foods with low atherogenic risk.

On the other hand, TI characterizes the thrombogenic potential of FA, indicating the tendency to form clots in blood vessels, and provides the contribution of different FA, which denotes the relationship between a pro- (C12:0, C14:0, and C16:0) and anti-thrombogenic (MUFA and the n-3 and n-6 PUFA) FA [21]. Therefore, the consumption of foods with a low TI is beneficial for avoiding cardiovascular disorders. We found better TI values for horse fat and meat, lard, sole, and duck egg.

However, in our opinion, the AI and TI do not consider the complexity of the fatty acid profile, nor the fat content; thus, they lack accuracy.

In agreement to what was previously noted for the TI and IA, the best HFI results were found for low-fat horse meat, lard, sole, gorgonzola, and duck egg. Nevertheless, no concerns about the low-fat horse and sole were indicated, the lard represented unexpected results.

The present results, demonstrated that for developing an index that describe the “healthy/non-healthy” foods for human nutrition should also consider the recommended dose. A deeper knowledge of the whole acidic composition of lipids of animal origin is seminal for a tailored, personalized nutrition beyond a generic demonization of certain food considered unhealthy.

In conclusion, it can be stated that the findings of this investigation pointed out the need to better characterize foods from a lipidic point of view; it is desirable to consider the lipid, but, at the same time, the fatty acid profile should be carefully evaluated in order to precisely define the recommended daily dose, avoiding generalizing only by food class (meat, processed foods, fish, cheeses, etc.). Obviously, the HFI can be improved, and this proposal aims to represent a starting point for researchers who study in this area in order to define increasingly appropriate indices for their own scientific purposes. It is clear that all indices exhibit advantages and disadvantages; therefore, a rational choice should be applied to consider the nutritional effect of foods on human health, as well as for possible evaluations regarding the methods for producing such foods (genetics, production systems, distribution, packaging, cooking, etc.).

Future efforts regarding this topic will certainly include a careful study aimed at refining the weights of the different fatty acid classes (or of the single fatty acid), incorporating the recommended doses in the HFI formula for its final testing in humans through employing clinical trials, and including enriched or functional foods.

## Figures and Tables

**Table 1:** Agreement between indexes: results of Intraclass correlation coefficients (ICC).

Agreement *	HFI Level	Intraclass Correlation	95% Confidence Interval		Sig.
			Lower Bound	Upper Bound	
HFI-AI	High HFI	0.193	−0.162	0.550	0.152
	Medium HFI	0.032	−0.140	0.318	0.388
	Low HFI	0.344	−0.139	0.688	0.076
	All data	0.505	0.277	0.679	<0.001
HFI-TI	High HFI	0.408	−0.054	0.723	0.041
	Medium HFI	0.101	−0.067	0.392	0.055
	Low HFI	0.465	0.055	0.748	0.010
	All data	0.778	0.647	0.864	<0.001
AI-TI	High HFI	0.112	−0.109	0.417	0.152
	Medium HFI	−0.120	−0.557	0.368	0.684
	Low HFI	0.779	0.487	0.911	<0.001
	All data	0.783	0.655	0.868	<0.001

* Calculated after standardization. Sig.: significance; −: minus. Agreement was poor if ICC < 0.40, fair if 0.40 ≤ ICC < 0.60, good if 0.60 ≤ ICC < 0.75, and excellent if ICC ≥ 0.75.

**Table 2:** Agreement in the classification of foods according to the different indices: the McNemar—Bowker test and Cohen’s kappa.

Agreement	McNemar–Bowker Test		Cohen’s Kappa	
	Value	*p* Value *	Value ^†^	*p* Value ^#^
HFI-AI opposite	0.200	0.978	0.373	<0.001
HFI-TI opposite	0.200	0.905	0.754	<0.001
AI-TI	3.686	0.297	0.263	0.006

*—Asymptotic significance (two-sided); ^†^ agreement was poor if k < 0.00, slight if 0.00 ≤ k ≤ 0.20, fair if 0.21 ≤ k ≤ 0.40, moderate if 0.41 ≤ k ≤ 0.60, substantial if 0.61 ≤ k ≤ 0.80, almost perfect if k > 0.80. ^#^—exact significance.

**Table 3:** Nutritional composition, energy, and indices of meats and cuts of different animals species.

	Portion (g)	Lipids (g/100 g)	Energy (Kcal)	Healthy Fatty Index	Atherogenic Index	Thrombogenic Index
Beef						
Fillet	100	5.0	127	2.87	0.50	0.85
Rump	100	2.3	106	2.91	0.50	0.83
Loin	100	5.2	134	2.94	0.49	0.82
Pork						
Loin	100	7.0	146	2.99	0.40	1.05
Shoulder	100	8.0	157	2.63	0.62	1.24
Steak	100	6.3	133	2.00	0.85	1.68
Lamb						
Thigh	100	2.5	102	2.00	0.56	1.49
Chop	100	2.7	106	1.92	0.78	1.54
Horse						
Low-fat meat	100	1.0	106	4.04	0.41	0.91
Fat and meat	100	6.8	143	3.00	0.65	0.76
Chicken						
Whole with skin	100	10.6	171	2.85	0.48	0.98
Breast standard	100	0.8	100	3.76	0.41	0.77
Breast SG	100	0.25	75	3.22	0.55	0.94
Breast FG	100	1.45	125	2.80	0.64	1.24
Turkey breast	100	1.2	107	3.24	0.51	0.91
Rabbit loin	100	1.0	105	3.28	0.68	0.87

Green text: the best food; red text: the worst food. Highlighted green/red numbers indicate values very close to those in green/red text.

**Table 4:** Nutritional composition, energy, and estimated indices of processed meat.

	Portion (g)	Lipids (g/100 g)	Energy (Kcal)	Healthy Fatty Index	Atherogenic Index	Thrombogenic Index
Crude Italian ham	50 g	18.6	271	2.11	0.82	1.40
Cooked ham	50 g	7.6	138	1.81	0.92	1.63
Speck	50 g	19.1	299	2.36	0.74	1.21
Bresaola	50 g	2.0	152	2.60	0.41	1.08
Salami	50 g	28.5	370	2.58	0.44	1.01
Pork lard	10 g	99.0	981	3.44	0.36	0.81
Mortadella	50 g	25.0	288	2.59	1.04	1.13
Pure pork Wurstel	50 g	21.1	250	2.39	0.46	1.10
Suet	10 g	99.0	892	2.05	0.54	1.41

Green text: the best food; red text: the worst food. Highlighted green/red numbers indicate values very close to those in green/red text.

**Table 5:** Nutritional composition, energy, and estimated indices of fish/shellfish/crustaceans.

	Portion (g)	Lipids (g/100 g)	Energy (Kcal)	Healthy Fatty Index	Atherogenic Index	Thrombogenic Index
Seabream bass						
Caught	150	3.8	121	4.47	0.53	0.45
Farmed	150	8.4	159	6.11	0.48	0.27
Sea bas fillet						
Caught	150	1.5	82	5.65	0.38	0.32
Farmed	150	6.8	149	5.30	0.45	0.31
Cod						
Fresh	150	0.9	71	9.62	0.26	0.14
Frozen	150	0.6	75	9.00	0.38	0.18
Sole	150	1.4	83	14.07	0.23	0.09
Salmon						
Fresh	150	12	185	4.89	0.54	0.33
Smoked	150	4.5	147	4.83	0.53	0.33
Tuna	150	8.1	159	3.69	0.84	0.61
Farmed eel	150	18.9	237	4.55	0.53	0.38
Anchovy	150	2.5	96	4.57	1.26	0.48
Mussel	150	2.7	84	5.67	0.40	0.31
Clams	150	1.2	65	6.85	0.58	0.26
Frozen shelled prawns	150	0.9	85	7.59	0.56	0.21
Lobster	150	1.9	89	7.68	0.31	0.12
Octopus	150	1.0	57	8.59	0.73	0.13

Green text: the best food; red text: the worst food. Highlighted green numbers indicate values very close to those in green text.

**Table 6:** Nutritional composition, energy, and estimated indices of milk and milk products.

	Portion (g)	Lipids (g/100 g)	Energy (Kcal)	Healthy Fatty Index	Atherogenic Index	Thrombogenic Index
Aged cheese	50	29.7	397	0.93	2.36	2.54
Mixed caciotta	50	31.0	192	1.17	2.34	2.10
Cow ricotta	100	10.9	144	0.92	2.92	3.05
Sheep ricotta	100	11.5	157	1.04	2.94	2.07
Creamy Blue cheese	50	27.1	324	2.95	1.97	0.64
Yogurt from whole milk	125	3.9	278	0.95	2.68	2.65
Whole milk	125	3.6	63	1.07	2.06	2.29
Semi-skimmed milk	125	1.5	46	1.21	2.15	2.37
Butter	10	83.4	758	1.04	2.13	2.38

Green text: the best food; red text: the worst food. Highlighted green/red numbers indicate values very close to those in green/red text.

**Table 7:** Nutritional composition, energy, and estimated indices of Eeggs.

	Portion (g)	Lipids (g/100 g)	Energy (Kcal)	Healthy Fatty Index	Atherogenic Index	Thrombogenic Index
Chicken egg	50	8.7	128	2.16	0.53	1.56
Yolk, conventional	15	29.7	325	2.34	0.49	1.47
Yolk, bio-plus	15	29.1	310	2.99	0.33	0.64
Duck egg	50	15.4	190	3.29	0.34	0.74

Green text: the best food; red text: the worst food. Highlighted green numbers indicate values very close to those in green text.

## Data Availability

Data are available in a publicly accessible repository. The data presented in this study are openly available on the CREA (Food and Nutrition Research Center) website (https://www.alimentinutrizione.it/tabelle-nutrizionali/ricerca-per-alimento, accessed on 1 April 2023).

## Supplementary Materials

The following supporting information can be downloaded at: https://www.mdpi.com/article/10.3390/foods13101568/s1, Table S1. Classification of foods according to the HFI value categorized by the binning technique; Table S2. Classification of foods according to the AI value categorized by the binning technique; Table S3. Classification of foods according to the AI value categorized by the binning technique; Table S4. Crosstabulation of HFI and AI categories. Table S5. Crosstabulation of HFI and TI categories. Table S6. Crosstabulation of AI and TI categories. Table S7. Results of the automatic linear modeling procedures, including the indices (HFI, IA, IT) as target and the percentage of SFA and n-3 as predictors. Table S8. Fatty acids profile (% of total FA) of meat sub-category; Table S9 Fatty acids profile (mg/100 g) of meat sub-category; Table S10. Fatty acids profile (% of total FA) of processed meat sub-category; Table S11. Fatty acids profile (mg/100 g) of processed meat sub-category. Table S12. Fatty acids profile (% of total FA) of fish/shellfish/crustaceans sub-category. Table S13. Fatty acids profile (mg/100 g) of fish/shellfish/crustaceans sub-category. Table S14. Fatty acids profile (% of total FA) of cheeses sub-category. Table S15. Fatty acids profile (mg/100 g) of cheeses sub-category. Table S16. Fatty acids profile (% of total FA) of eggs sub-category. Table S17. Fatty acids profile (mg/100 g) of eggs sub-category.

## References

[1] Calder P.C. (2015). Functional roles of fatty acids and their effects on human health. J. Parenter. Enter. Nutr..

[2] Sanders T.A.B. (2010). The role of fat in the diet–quantity, quality and sustainability. Nutr. Bull..

[3] Sanders T.A.B. (2024). Introduction: The role of fats in human diet. Funct. Diet. Lipids.

[4] Marventano S., Kolacz P., Castellano S., Galvano F., Buscemi S., Mistretta A., Grosso G. (2015). A review of recent evidence in human studies of n-3 and n-6 PUFA intake on cardiovascular disease, cancer, and depressive disorders: Does the ratio really matter?. Int. J. Food Sci. Nutr..

[5] Akonjuen B.M., Onuh J.O., Aryee A.N.A. (2023). Bioactive fatty acids from non-conventional lipid sources and their potential application in functional food development. Food Sci. Nutr..

[6] Gebauer S., Harris W.S., Kris-Etherton M., Etherton T.D. (2019). Dietary n-6: N-3 fatty acid ratio and health. Healthful Lipids.

[7] Vaezi M. (2023). Efficacy and Biomedical Roles of Unsaturated Fatty Acids as Bioactive Food Components. Curr. Chem. Biol..

[8] Lopez A., Bellagamba F., Moretti V.M. (2024). Fatty Acids. Handbook of Seafood and Seafood Products Analysis.

[9] Horman T., Fernandes M.F., Tache M.C., Hucik B., Mutch D.M., Leri F. (2020). Dietary N-6/N-3 ratio influences brain fatty acid composition in adult rats. Nutrients.

[10] Sokoła-Wysoczańska E., Wysoczański T., Wagner J., Czyż K., Bodkowski R., Lochyński S., Patkowska-Sokoła B. (2018). Polyunsaturated fatty acids and their potential therapeutic role in cardiovascular system disorders—A review. Nutrients.

[11] Dréano-Trécant L., Egnell M., Hercberg S., Galan P., Soudon J., Fialon M., Touvier M., Kesse-Guyot E., Julia C. (2020). Performance of the front-of-pack nutrition label Nutri-Score to discriminate the nutritional quality of foods products: A comparative study across 8 European countries. Nutrients.

[12] Hau R.C., Lange K.W. (2023). Can the 5-colour nutrition label “Nutri-Score” improve the health value of food?. J. Futur. Foods.

[13] Tachie C., Tawiah N.A., Aryee A.N.A. (2023). Current Research in Food Science. Curr. Res. Food Sci..

[14] Dal Bosco A., Mancinelli A.C., Vaudo G., Cavallo M., Castellini C., Mattioli S. (2022). Indexing of Fatty Acids in Poultry Meat for Its Characterization in Healthy Human Nutrition: A Comprehensive Application of the Scientific Literature and New Proposals. Nutrients.

[15] Mattioli S., Castellini C., Mancini S., Roscini V., Mancinelli A.C., Cotozzolo E., Pauselli M., Dal Bosco A. (2020). Effect of trub and/or linseed dietary supplementation on in vivo oxidative status and some quality traits of rabbit meat. Meat Sci..

[16] Mugnai C., Sossidou E.N., Dal Bosco A., Ruggeri S., Mattioli S., Castellini C. (2014). The effects of husbandry system on the grass intake and egg nutritive characteristics of laying hens. J. Sci. Food Agric..

[17] Luca S., Laura C., Maurizio M., Claudio M., Pierluigi P., Angela P., Andrea S., Anna T., Furio B., Rita A. (2014). LARN Livelli di Assunzione di Riferimento di Nutrienti ed Energia per la Popolazione Italiana. IV Revisione.

[18] Centro di Ricerca Alimenti e Nutrizione Linee Guida Per Una Sana Alimentazione. Univesità degli Studi di Parma 2019. On Line Report. ISBN: 9788833850375. https://www.salute.gov.it/imgs/C_17_pubblicazioni_2915_allegato.pdf.

[19] Greenfield H., Southgate D.A.T. (2003). Food Composition Data: Production, Management, and Use.

[20] Weihrauch J.L., Posati L.P., Anderson B.A., Exler J. (1977). Lipid conversion factors for calculating fatty acid contents of foods. J. Am. Oil Chem. Soc..

[21] Ulbricht T.L.V., Southgate D.A.T. (1991). Coronary heart disease: Seven dietary factors. Lancet.

[22] Omri B., Chalghoumi R., Izzo L., Ritieni A., Lucarini M., Durazzo A., Abdouli H., Santini A. (2019). Effect of dietary incorporation of linseed alone or together with tomato-red pepper mix on laying hens’ egg yolk fatty acids profile and health lipid indexes. Nutrients.

[23] Calder P.C. (2006). Polyunsaturated fatty acids and inflammation. Prostaglandins Leukot. Essent. Fat. Acids.

[24] Mansournia M.A., Waters R., Nazemipour M., Bland M., Altman D.G. (2021). Bland-Altman methods for comparing methods of measurement and response to criticisms. Glob. Epidemiol..

[25] Menchetti L., Zappaterra M., Nanni Costa L., Padalino B. (2021). Application of a protocol to assess camel welfare: Scoring system of collected measures, aggregated assessment indices, and criteria to classify a pen. Animals.

[26] Fleiss J.L. (2011). Design and Analysis of Clinical Experiments.

[27] Watson P.F., Petrie A. (2010). Method agreement analysis: A review of correct methodology. Theriogenology.

[28] Erlanson-Albertsson C. (2010). Fat-rich food palatability and appetite regulation. Fat Detection: Taste, Texture, and Post Ingestive Effects.

[29] Lenighan Y.M., McNulty B.A., Roche H.M. (2019). Dietary fat composition: Replacement of saturated fatty acids with PUFA as a public health strategy, with an emphasis on α-linolenic acid. Proc. Nutr. Soc..

[30] Wood J.D., Richardson R.I., Nute G.R., Fisher A.V., Campo M.M., Kasapidou E., Sheard P.R., Enser M. (2004). Effects of fatty acids on meat quality: A review. Meat Sci..

[31] Covington M.B. (2004). Omega-3 fatty acids. Am. Fam. Physician.

[32] Givens D.I. (2024). Modification of the fatty acid composition of animal-derived foods: Does it enhance their healthiness?. Functional Dietary Lipids.

[33] Savatinova M., Ivanova M. (2024). Functional dairy products enriched with omega-3 fatty acids. Food Sci. Appl. Biotechnol..

[34] Willett W.C. (2012). Dietary fats and coronary heart disease. J. Intern. Med..

[35] Brukało K.M., Nowak J., Pietrzykowska A., Fras N., Ožbolt P., Kowalski O., Blenkuš M.G. (2024). Public food procurement as a tool of sustainable food and nutrition policy—Fat products. Front. Sustain. Food Syst..

[36] Lescinsky H., Afshin A., Ashbaugh C., Bisignano C., Brauer M., Ferrara G., Hay S.I., He J., Iannucci V., Marczak L.B. (2022). Health effects associated with consumption of unprocessed red meat: A Burden of Proof study. Nat. Med..

[37] Chen J., Liu H. (2020). Nutritional indices for assessing fatty acids: A mini-review. Int. J. Mol. Sci..

[38] Grosso G. (2023). Role of food processing on human health and current limitations. Int. J. Food Sci. Nutr..

[39] Soladoye O.P., Juárez M.L., Aalhus J.L., Shand P., Estévez M. (2015). Protein oxidation in processed meat: Mechanisms and potential implications on human health. Compr. Rev. Food Sci. Food Saf..

[40] Lands W.E.M. (1992). Biochemistry and physiology of n-3 fatty acids. FASEB J..

[41] Tunick M.H., Van Hekken D.L. (2015). Dairy products and health: Recent insights. J. Agric. Food Chem..

[42] Thorning T.K., Raben A., Tholstrup T., Soedamah-Muthu S.S., Givens I., Astrup A. (2016). Milk and dairy products: Good or bad for human health? An assessment of the totality of scientific evidence. Food Nutr. Res..

[43] Réhault-Godbert S., Guyot N., Nys Y. (2019). The golden egg: Nutritional value, bioactivities, and emerging benefits for human health. Nutrients.

[44] Fernández-reiriz M.J., Irisarri J., Labarta U. (2017). Fatty acid composition of female and male clams (Ruditapes philippinarum): Energy intake and temperature reliance. Aquac. Nutr..

[45] Mugnai C., dal Bosco A., Castellini C. (2009). Effect of rearing system and season on the performance and egg characteristics of Ancona laying hens. Ital. J. Anim. Sci..

