# Supplementary material for: The Healthy Fatty Index Allows for Deeper Insights into the Lipid Composition of Foods of Animal Origin When Compared with the Atherogenic and Thrombogenicity Indexes
Source: Foods. 2024 May 17;13(10):1568. doi: 10.3390/foods13101568 (PMC11120502; doi:10.3390/foods13101568)
Supplement: Supplementary file 1 [file foods-13-01568-s001.zip › foods-2986224-supplementary.pdf]

Supplementary tables

Table S1. Classification of foods according to the HFI value categorized by the binning technique

**HFI (Binned)**		
**Low HFI**<br>**(<= 2.575)**	**Medium HFI**<br>**(2.576 - 3.685)**	**High HFI**<br>**(3.6854+)**
Aged cheese	Beef Fillet	Anchovy
Butter	Beef Sirloin steak	Chicken Breast standard
Chicken egg	Beef Thick flank	Clams
Cooked ham	Blue cheese	Cod Fresh
Cow ricotta	Bresaola	Cod Frozen
Crude Italian ham	Chicken Breast FG	Farmed Eel
Lamb Cutlet	Chicken Breast SG	Frozen shelled prawns
Lamb Thigh	Chicken Whole with skin	Horse Low-fat meat
Mixed caciotta	Duck egg	Lobster
Pork Steak	Horse Fat and meat	Mussel
Pure pork Wurstel	Mortadella	Octopus
Salami	Pork lard	Salmon fresch
Semi-skimmed milk	Pork Loin	Salmon smoked
Sheep ricotta	Pork Shoulder	Sea bas fillet Caught
Speck	Rabbit loin	Sea bas fillet Farmed
Suet	Turkey breast	Seabream bass Caught
whole milk	Yolk Bio-plus	Seabream bass Farmed
Yogurt from whole milk		Sole
Yolk conventional		Tuna

Table S2. Classification of foods according to the AI value categorized by the binning technique

AI (Binned)		
**Low AI** (<= 0.49)	**Medium AI** (0.491 - 0.679)	**High AI** (0.6789+)
Bresaola	Beef Fillet	Aged cheese
Chicken Breast standard	Beef Sirloin steak	Anchovy
Chicken Whole with skin	Beef Thick flank	Blue cheese
Cod fresh	Chicken breast fg	Butter
Cod frozen	Chicken breast sg	Cooked ham
Duck egg	Chicken egg	Cow ricotta
Horse Low-fat meat	Clams	Crude Italian ham
Lobster	Farmed Eel	Lamb Cutlet
Mussel	Frozen shelled prawns	Mixed caciotta
Pork lard	Horse Fat and meat	Mortadella
Pork loin	Lamb thigh	Octopus
Pure pork Wurstel	Pork Shoulder	Pork Steak
Salami	Rabbit loin	Semi-skimmed milk
Sea bas fillet Farmed	Salmon fresch	Sheep ricotta
Seabream bass Caught	Salmon smoked	Speck
Seabream bass Farmed	Sea bas fillet Caught	Tuna
Sole	Suet	Whole milk
Yolk Bio-plus	Turkey breast	Yogurt from whole milk
Yolk conventional		

Table S3. Classification of foods according to the TI value categorized by the binning technique.

TI (Binned)		
**Low TI** (<= 0.670)	**Medium TI** (0.671 - 1.211)	**High TI** (1.211+)
Anchovy	Beef Fillet	Aged cheese
Blue cheese	Beef Sirloin steak	Butter
Clams	Beef Thick flank	Chicken Breast FG
Cod fresh	Bresaola	Chicken egg
Cod frozen	Chicken breast sg	Cooked ham
Farmed eel	Chicken breast standard	Cow ricotta
Frozen shelled prawns	Chicken Whole with skin	Crude Italian ham
Lobster	Duck egg	Lamb Cutlet
Mussel	Horse Fat and meat	Lamb Thigh
Octopus	Horse Low-fat meat	Mixed caciotta
Salmon fresch	Mortadella	Pork Shoulder
Salmon smoked	Pork lard	Pork Steak
Sea bas fillet Caught	Pork Loin	Semi-skimmed milk
Sea bas fillet Farmed	Pure pork Wurstel	Sheep ricotta
Seabream bass Caught	Rabbit loin	Suet
Seabream bass Farmed	Salami	Whole milk
Sole	Speck	Yogurt from whole milk
Tuna	Turkey breast	Yolk conventional
Yolk Bio-plus		

Table S4. Crosstabulation of HFI and AI categories.

AI* (Binned)	HFI (Binned)			Total
	Low HFI	Medium HFI	High HFI	
**Low AI (opposite)**	13 (68.4%)	2 (11.8%)	3 (15.8%)	18 (33.3%)
**Medium AI (opposite)**	3 (15.8%)	9 (52.9%)	6 (31.6%)	18 (33.3%)
**High AI (opposite)**	3 (15.8%)	6 (35.3%)	10 (52.6%)	19 (33.3%)
**Total**	19 (100.0%)	17 (100.0%)	19 (100.0%)	54 (100.0%)

* the opposite of AI was calculated to make the categories comparable
P value for McNemar-test=0.978

Table S5. Crosstabulation of HFI and TI categories.

TI* (Binned)	HFI (Binned)			Total
	Low HFI	Medium HFI	High HFI	
**Low TI (opposite)**	16 (84.2%)	2 (11.8%)	0 (0.0%)	18 (33.3%)
**Medium TI (opposite)**	3 (15.8%)	13 (76.5%)	2 (10.5%)	18 (33.3%)
**High TI (opposite)**	0 (0.0%)	2 (11.8%)	17 (89.5%)	19 (33.3%)
**Total**	19 (100.0%)	17 (100.0%)	19 (100.0%)	54 (100.0%)

* the opposite of AI was calculated to make the categories comparable
P value for McNemar-test=0.905

Table S6. Crosstabulation of AI and TI categories.

TI (Binned)	AI (Binned)			Total
	Low AI	Medium AI	High AI	
**Low TI**	9 (47.4%)	6 (33.3%)	4 (22.2%)	19 (33.3%)
**Medium TI**	9 (47.4%)	7 (38.9%)	2 (11.1%)	18 (33.3%)
**High TI**	1 (5.3%)	5 (27.8%)	12 (66.7%)	18 (33.3%)
**Total**	19 (100.0%)	18 (100.0%)	18 (100.0%)	54 (100.0%)

P value for McNemar-test=0.297

Table S7. Results of the Automatic Linear Modeling procedures including the indices (HFI: a), IA: b), IT: c)) as target and the percentage of SFA and n-3 as predictors.

a) Coefficients target: HFI

Model term	Coefficient	Standard error	t	Sig.	95% Confidence interval		Importance
					Lower	Upper	
Intercept	4.475	0.544	8.222	<0.001	3.3383	5.567	
n-3 transformed	0.146	0.011	13.010	<0.001	0.124	0.169	0.872
SFA transformed	-0.056	0.011	-4.978	<0.001	-0.078	-0.033	0.128

b) Coefficients target: IA

Model term	Coefficient	Standard error	t	Sig.	95% Confidence interval		Importance
					Lower	Upper	
Intercept	-1.645	0.163	-10.078	<0.001	-1.973	-1.318	
n-3 transformed	0.058	0.003	17.157	<0.001	0.051	0.064	0.948
SFA transformed	0.014	0.003	4.028	<0.001	0.007	0.020	0.052

c) Coefficients target: IT

Model term	Coefficient	Standard error	t	Sig.	95% Confidence interval		Importance
					Lower	Upper	
Intercept	-0.370	0.204	-1.810	0.076	-0.779	0.040	
n-3 transformed	0.039	0.004	9.194	<0.001	0.030	0.047	0.842
SFA transformed	-0.017	0.004	-3.984	<0.001	-0.025	-0.008	0.158

t: t-statistic value; Sig.: Significance

Table S8. Fatty acids profile (% of total FA) of meat sub-category.

	% of total FA															
	Beef			Pork			Lamb		Horse		Chicken				Turkey breast	Rabbit loin
	Fillet	Thick flank	Sirloin steak	Loin	Shoulder	Steak	Thigh	Cutlet	Low-fat meat	Fat and meat	Whole with skin	Breast standard	Breast SG	Breast FG		
C12:0	0.00	0.00	0.00	0.16	0.17	3.03	0.45	0.42	0.00	0.16	0.00	0.00	0.20	0.62	0.00	0.00
C14:0	2.56	2.53	2.46	1.10	2.27	4.21	3.64	3.79	1.27	3.88	1.33	1.49	0.71	1.04	2.94	2.40
C16:0	20.23	20.22	20.13	22.14	28.43	25.00	24.55	24.41	21.52	26.66	25.43	19.83	27.98	30.01	20.59	29.70
C18:0	13.95	14.16	14.09	11.46	8.21	14.87	14.09	14.31	11.39	3.72	9.62	14.88	12.02	11.00	12.75	7.00
Others	2.10	2.02	2.01	0.15	0.62	1.05	3.18	2.95	0.00	0.49	0.07	0.00	2.49	2.07	0.97	2.60
Total SFA	38.84	38.93	38.69	35.01	39.70	48.16	45.91	45.88	34.18	34.91	36.45	36.20	43.40	44.74	37.25	41.70
C14:1n-6	1.86	2.02	1.79	0.00	0.00	0.16	0.00	0.00	0.00	0.00	0.00	1.49	0.15	0.11	0.98	0.15
C16:1n-7	3.02	3.03	2.91	1.57	2.27	0.58	1.82	1.68	1.27	10.34	5.93	1.50	0.61	1.39	2.94	1.40
C18:1n-9	29.30	29.32	29.29	32.97	32.44	18.46	36.82	36.62	13.92	34.26	34.38	23.80	19.50	23.04	24.51	24.40
Others	3.73	3.55	3.80	2.82	2.27	0.28	0.45	1.26	0.00	2.10	1.85	1.48	0.26	0.17	1.96	0.28
Total MUFA	37.91	37.92	37.79	37.36	36.98	32.76	39.09	39.56	15.19	46.70	42.16	28.27	20.52	23.94	30.39	26.00
C18:2n-6	10.93	11.12	10.96	26.37	23.55	19.08	10.93	9.68	31.65	12.12	16.68	20.50	19.09	20.70	18.63	24.10
C20:2n-6	0.00	0.00	0.00	0.00	0.00	0.00	0.00	0.00	0.00	0.00	0.00	0.00	0.68	0.20	0.00	0.29
C20:3n-6	0.00	0.00	0.00	0.00	0.00	0.00	0.00	0.00	0.00	0.00	0.00	0.00	0.17	0.16	0.00	0.00
C20:4n-6	3.26	3.03	3.13	0.47	0.52	0.00	4.09	3.37	7.59	1.13	2.56	8.40	10.60	8.03	6.86	0.43

Total n-6	14.19	14.15	14.09	26.84	24.07	19.08	15.02	13.05	39.24	13.25	19.24	28.90	29.54	29.09	25.49	24.82
C18:3n-3	2.09	2.02	2.24	0.32	0.29	0.13	0.45	0.42	2.24	5.01	1.23	5.20	2.03	0.98	0.98	1.00
C20:3n-3	0.00	0.00	0.00	0.00	0.03	0.00	0.00	0.00	0.00	0.00	0.00	0.00	0.04	0.03	0.00	0.00
C20:5n-3	2.56	2.53	2.68	0.00	0.00	0.00	0.00	0.00	1.27	0.00	0.20	0.50	0.68	0.09	0.00	5.40
C21:5n-3	0.00	0.00	0.00	0.00	0.00	0.00	0.00	0.00	0.00	0.00	0.00	0.00	0.87	0.03	0.00	0.00
C22:5n-3	0.00	0.00	0.00	0.00	0.00	0.00	0.00	0.00	0.00	0.00	0.00	0.00	1.37	0.92	0.00	0.00
C22:6n-3	1.06	1.52	1.34	0.00	0.00	0.00	0.00	0.00	0.00	0.00	0.72	0.50	1.05	0.18	2.94	0.10
Total n-3	5.71	6.07	6.26	0.32	0.32	0.13	0.45	0.42	3.51	5.01	2.15	6.20	4.54	2.23	3.92	6.50
n-6/n-3	2.49	2.33	2.25	83.88	75.22	146.77	33.38	31.07	11.18	2.64	8.95	4.66	6.50	13.04	6.50	3.82
Others	3.12	3.04	2.68	1.41	0.00	0.53	0.44	0.42	7.88	0.49	0.00	35.10	0.50	0.00	3.92	0.58
Total PUFA	23.02	23.26	23.03	28.57	24.07	19.74	15.91	13.89	50.63	18.75	21.39	25.41	35.58	31.32	33.33	31.90

FA: fatty acids; SFA: saturated fatty acids; MUFA: monounsaturated fatty acids; PUFA: polyunsaturated fatty acids; SG: slow-growing; FG: fast-growing

Table S9 Fatty acids profile (mg/100g) of meat sub-category.

	mg/100g															
	Beef			Pork			Lamb		Horse		Chicken				Turkey breast	Rabbit loin
	Fillet	Thick flank	Sirloin steak	Loin	Shoulder	Steak	Thigh	Cutlet	Low-fat meat	Fat and meat	Whole with skin	Breast standard	Breast SG	Breast FG		
C12:0	0.00	0.00	0.00	9.69	9.27	209.77	9.74	9.81	0.00	9.42	0.00	0.00	0.43	7.41	0.00	0.00
C14:0	110.77	50.36	110.70	66.63	123.76	291.46	78.75	88.55	10.99	235.04	122.00	10.32	1.54	12.42	30.53	27.00
C16:0	875.33	402.45	905.84	1341.16	1549.96	1730.75	531.12	570.34	186.23	1614.96	2332.69	137.28	60.53	358.45	213.82	334.12
C18:0	603.60	281.84	634.04	694.20	447.60	1029.45	304.83	334.35	98.57	225.34	882.44	103.01	30.33	131.39	132.40	78.75
Others	90.86	40.21	90.45	9.09	33.80	72.69	68.80	68.93	0.00	29.68	6.42	0.00	7.55	24.72	10.07	29.25
Total SFA	1680.56	774.85	1741.03	2120.77	2164.39	3334.12	993.23	1071.99	295.79	2114.72	3343.55	250.61	100.38	534.39	386.82	469.12
C14:1n-6	80.48	40.21	80.55	0.00	0.00	11.08	0.00	0.00	0.00	0.00	0.00	10.32	0.32	1.31	10.18	1.69
C16:1n-7	130.67	60.31	130.95	95.10	123.76	40.15	39.37	39.25	10.99	626.36	543.96	10.38	1.32	16.60	30.53	15.75
C18:1n-9	1267.77	583.57	1318.04	1997.20	1768.58	1277.99	796.58	855.63	120.46	2075.34	3153.67	164.77	40.02	275.20	254.52	274.50
Others	161.39	70.66	171.00	170.83	123.76	19.38	9.74	29.44	0.00	127.21	169.70	10.25	0.56	2.03	20.35	3.15
Total MUFA	1640.32	754.75	1700.53	2263.13	2016.10	2267.97	845.69	924.32	131.45	2828.91	3867.33	195.71	42.23	293.95	315.58	292.50
C18:2n-6	472.93	221.33	493.19	1597.40	1283.91	1320.91	236.46	226.17	273.89	734.18	1530.05	141.92	39.14	247.25	193.46	271.12
C20:2n-6	0.00	0.00	0.00	0.00	0.00	0.00	0.00	0.00	0.00	0.00	0.00	0.00	1.47	2.39	0.00	3.26
C20:3n-6	0.00	0.00	0.00	0.00	0.35	0.00	0.00	0.00	0.00	0.00	0.00	0.00	0.37	1.91	0.00	0.00

C20:4n-6	141.06	60.31	140.85	28.47	28.35	0.00	88.48	78.74	65.68	68.45	234.83	58.15	22.93	95.91	71.24	4.84
Total n-6	613.98	281.64	634.04	1625.87	1312.26	1320.91	324.95	304.91	339.57	802.64	1764.88	200.07	63.91	347.46	264.70	279.22
C18:3n-3	90.43	40.21	100.80	19.38	15.81	9.00	9.74	9.81	19.38	303.49	112.83	36.00	2.23	11.71	10.18	11.25
C20:3n-3	0.00	0.00	0.00	0.00	1.64	0.00	0.00	0.00	0.00	0.00	0.00	0.00	0.09	0.36	0.00	0.00
C20:5n-3	110.77	50.36	120.60	0.00	0.00	0.00	0.00	0.00	10.99	0.00	18.35	3.46	0.39	1.07	0.00	60.75
C21:5n-3	0.00	0.00	0.00	0.00	0.00	0.00	0.00	0.00	0.00	0.00	0.00	0.00	1.88	0.36	0.00	0.00
C22:5n-3	0.00	0.00	0.00	0.00	0.00	0.00	0.00	0.00	0.00	0.00	0.00	0.00	2.96	10.99	0.00	0.00
C22:6n-3	45.86	30.25	60.30	0.00	0.00	0.00	0.00	0.00	0.00	0.00	66.05	3.46	2.26	2.15	30.53	1.12
Total n-3	247.06	120.82	281.70	19.38	17.45	9.00	9.74	9.81	30.37	303.49	197.22	42.92	9.81	26.64	40.71	73.12
n-6/n-3	2.49	2.33	2.25	83.88	75.22	146.77	33.38	31.07	11.18	2.64	8.95	4.66	6.51	13.04	6.50	3.82
Total PUFA	996.05	462.96	1036.34	1730.66	1312.26	1366.60	688.41	276.46	438.14	1135.80	1962.10	243.00	73.72	374.10	346.12	358.87
Total	4316.93	1992.56	4477.9	6114.56	5492.75	6968.69	2527.33	2272.77	865.38	6079.43	9172.98	689.32	216.33	1202.44	1048.52	1120.49

FA: fatty acids; SFA: saturated fatty acids; MUFA: monounsaturated fatty acids; PUFA: polyunsaturated fatty acids; SG: slow-growing; FG: fast-growing

Table S10. Fatty acids profile (% of total FA) of processed meat sub-category.

% of total FA									
	**Crude Italian ham**	**Cooked ham**	**Speck**	**Bresaola**	**Salami**	**Pork Lard**	**Mortadella**	**Pure pork Wurstel**	**Suet**
C12:0	0.10	0.20	0.30	0.00	0.10	0.07	0.10	0.10	0.24
C14:0	6.50	6.50	5.80	1.40	1.64	0.63	10.50	1.40	1.36
C16:0	21.10	22.90	20.30	17.90	21.85	21.28	19.10	21.80	24.76
C18:0	14.90	16.60	14.00	12.80	11.94	11.20	10.20	11.50	17.18
Others	0.60	0.40	0.60	0.90	0.09	0.27	1.60	0.60	0.23
Total SFA	43.20	46.60	41.00	33.00	35.62	33.45	41.50	35.40	43.77
C14:1n-6	0.00	0.00	0.00	0.15	0.00	0.00	0.00	0.00	0.52
C16:1n-7	2.60	2.60	2.70	2.30	2.10	1.30	2.70	2.80	2.63
C18:1n-9	40.30	40.00	38.90	27.90	44.81	33.97	42.10	42.30	40.26
Others	1.10	0.90	1.10	0.28	2.52	2.25	1.20	1.10	1.02
Total MUFA	44.00	43.50	42.70	31.60	49.43	37.52	46.00	46.20	44.43
C18:2n-6	12.10	8.70	13.70	9.60	14.34	26.09	20.70	12.00	9.22
C20:2n-6	0.00	0.00	0.00	0.00	0.00	0.00	0.00	0.00	0.00
C20:3n-6	0.00	0.00	0.00	0.00	0.00	0.00	0.00	0.00	0.00
C20:4n-6	1.00	0.50	1.30	4.10	0.00	0.00	0.40	0.50	1.89
Total n-6	13.10	9.20	15.00	13.70	14.34	26.09	21.10	12.50	11.11
C18:3n-3	0.60	0.60	1.10	2.30	0.80	2.97	0.60	0.70	0.95
C20:3n-3	0.00	0.00	0.00	0.00	0.00	0.00	0.00	0.00	0.00

C20:5n-3	0.00	0.00	0.32	0.00	0.31	0.00	0.00	0.00	0.00
C21:5n-3	0.00	0.00	0.00	0.00	0.00	0.00	0.00	0.00	0.00
C22:5n-3	0.00	0.00	0.00	0.00	0.00	0.00	0.00	0.00	0.00
C22:6n-3	0.00	0.00	0.00	0.10	0.00	0.00	0.00	0.00	0.00
Total n-3	0.60	0.60	1.42	2.40	1.11	2.97	0.60	0.70	0.95
n-6/n-3	21.83	15.33	10.56	5.71	12.92	8.78	35.17	17.86	11.69
Total PUFA	13.70	9.80	16.10	26.00	15.45	29.06	12.70	13.20	12.06

FA: fatty acids; SFA: saturated fatty acids; MUFA: monounsaturated fatty acids; PUFA: polyunsaturated fatty acids.

Table S11. Fatty acids profile (mg/100g) of processed meat sub-category.

mg/100g									
	**Raw ham**	**Cooked ham**	**Speck**	**Bresaola**	**Salami**	**Pork Lard**	**Mortadella**	**Pure pork Wurstel**	**Suet**
C12:0	16.10	20.60	49.59	0.00	24.66	59.97	21.63	18.26	205.61
C14:0	1046.24	669.37	958.66	24.23	404.48	539.73	2271.61	255.63	1165.14
C16:0	3396.25	2358.23	3355.32	309.80	5388.91	18231.03	4132.17	3980.55	21212.42
C18:0	2398.30	1709.46	2314.01	221.54	2944.78	9595.28	2206.71	2099.83	14718.47
Others	96.58	41.19	99.17	15.58	22.20	231.31	346.15	109.56	197.05
Total SFA	6953.46	4798.85	6776.75	571.15	8785.03	28657.33	8978.27	6463.83	37498.69
C14:1n-6	0.00	0.00	0.00	2.60	0.00	0.00	0.00	0.00	445.50
C16:1n-7	418.50	267.75	446.27	39.81	517.93	1113.74	584.13	511.26	2253.18
C18:1n-9	6486.68	4119.19	6429.65	482.88	11051.57	29102.82	9108.07	7723.73	34491.60
Others	177.06	92.68	181.82	4.85	621.51	1927.62	259.61	200.85	873.86
Total MUFA	7082.23	4479.61	7057.74	546.92	12191.01	32144.18	9951.81	8435.85	38064.13
C18:2n-6	1947.61	895.92	2264.43	166.15	3536.70	22351.86	4478.32	2191.13	7898.97
C20:2n-6	0.00	0.00	0.00	0.00	0.00	0.00	0.00	0.00	0.00
C20:3n-6	0.00	0.00	0.00	0.00	0.00	0.00	0.00	0.00	0.00
C20:4n-6	160.96	51.49	214.87	70.96	0.00	0.00	86.54	91.30	1619.20
Total n-6	2108.57	947.41	2479.30	237.11	3536.70	22351.86	4564.85	2282.43	9518.17
C18:3n-3	96.58	61.79	181.82	39.81	197.31	2544.46	129.81	127.82	813.89

C20:3n-3	0.00	0.00	0.00	0.00	0.00	0.00	0.00	0.00	0.00
C20:5n-3	0.00	0.00	52.89	0.00	76.46	0.00	0.00	0.00	0.00
C21:5n-3	0.00	0.00	0.00	0.00	0.00	0.00	0.00	0.00	0.00
C22:5n-3	0.00	0.00	0.00	0.00	0.00	0.00	0.00	0.00	0.00
C22:6n-3	0.00	0.00	0.00	1.73	0.00	0.00	0.00	0.00	0.00
Total n-3	96.58	61.79	234.71	41.54	273.76	2544.46	129.81	127.82	813.89
n-6/n-3	21.83	15.33	10.56	5.71	12.92	8.78	35.17	17.86	11.69
Total PUFA	2205.15	1009.20	2661.11	450.00	3810.46	24896.32	2747.57	2410.24	10332.06
Total	16240.84	10287.66	16495.6	1568.07	24786.5	85697.83	21677.65	17309.92	85894.88

FA: fatty acids; SFA: saturated fatty acids; MUFA: monounsaturated fatty acids; PUFA: polyunsaturated fatty acids.

Table S12. Fatty acids profile (% of total FA) of Fish / Shellfish / Crustaceans sub-category.

	% of total FA																
	Seabream fillet		Sea bass fillet		Cod		Sole	Salmon		Tuna	Farmed Eel	Anchovy	Mussel	Clams	Frozen shelled prawns	Lobster	Octopus
	Caught	Farmed	Caught	Farmed	Fresh	Frozen		Fresh	Smoked								
C12:0	0.00	0.00	0.00	0.00	0.00	0.10	0.10	0.00	0.00	0.00	0.00	0.00	0.00	0.00	0.00	0.00	0.00
C14:0	1.33	3.34	2.86	5.56	0.02	2.31	2.44	4.92	4.80	3.83	5.50	5.49	2.89	3.92	5.56	0.00	3.96
C16:0	21.65	19.16	23.80	14.87	20.08	18.52	9.63	19.22	19.19	33.46	17.30	39.65	16.78	20.26	17.78	20.83	27.72
C18:0	5.66	5.08	6.67	3.10	1.00	4.63	2.44	3.98	4.04	3.83	3.31	5.10	10.42	5.14	5.56	7.13	9.90
Others	0.67	0.58	0.00	0.00	3.00	0.00	0.00	0.01	0.27	0.24	0.00	0.78	0.00	3.76	1.10	6.58	0.00
Total SFA	29.31	28.16	33.33	23.53	24.10	25.56	14.61	28.13	28.30	41.36	26.11	51.02	30.09	33.08	30.00	34.54	41.58
C14:1n-6	0.00	0.00	0.00	0.00	0.00	0.00	0.00	0.00	0.00	0.00	0.69	0.00	0.00	0.00	0.00	0.00	0.00
C16:1n-7	4.33	6.68	5.71	9.31	20.08	2.31	6.49	6.16	6.06	2.72	8.69	3.92	9.26	7.78	4.44	0.00	1.98
C18:1n-9	20.65	24.97	20.00	18.30	8.03	16.89	10.55	23.30	23.23	11.73	28.68	6.67	9.26	8.62	21.11	17.54	7.92
Others	6.00	8.71	0.28	7.19	0.00	6.30	4.88	14.10	14.14	0.28	7.38	5.11	17.94	1.34	4.45	10.42	9.90
Total MUFA	30.98	40.36	28.57	34.80	28.11	25.50	21.92	43.56	43.43	18.64	45.44	15.70	36.46	17.74	30.00	27.96	19.80
C18:2n-6	3.66	6.10	0.95	6.05	0.00	0.00	0.01	1.42	0.51	1.85	6.31	0.39	2.89	1.29	2.22	0.00	0.01
C20:2n-6	0.00	0.00	0.00	0.00	0.00	0.00	0.00	0.00	0.00	0.00	0.00	0.00	0.00	0.00	0.00	0.00	0.00
C20:3n-6	0.00	0.00	0.00	0.00	0.00	0.00	0.00	0.00	0.00	0.00	0.00	0.00	0.00	0.00	0.00	0.00	0.00
C20:4n-6	6.33	1.02	3.81	6.05	4.02	2.31	14.61	0.47	0.51	0.01	3.19	0.01	6.37	3.37	0.51	0.01	0.00
Total n-6	9.99	7.12	4.76	12.10	4.02	2.31	14.62	1.89	1.02	1.86	9.50	0.40	9.26	4.66	2.73	0.01	0.01

C18:3n-3	0.33	0.73	0.95	0.33	0.00	0.00	5.68	0.85	0.76	1.11	0.31	0.39	2.31	1.17	1.11	0.00	0.00
C20:3n-3	0.00	0.00	0.00	0.00	0.00	0.00	0.00	0.00	0.00	0.00	0.00	0.00	0.00	0.00	0.00	0.00	0.00
C20:5n-3	9.66	6.78	8.57	7.84	12.05	6.94	17.86	8.43	8.33	9.88	5.00	10.60	15.63	15.67	17.27	9.88	17.82
C21:5n-3	0.00	0.00	0.00	0.00	0.00	0.00	0.00	0.00	0.00	0.00	0.00	0.00	0.00	0.00	0.00	0.00	0.00
C22:5n-3	0.00	0.00	0.00	0.00	0.00	0.00	0.00	0.00	0.00	0.00	0.00	0.00	0.00	0.00	0.00	0.00	0.00
C22:6n-3	12.66	13.79	0.10	12.65	32.13	34.72	25.97	11.27	11.36	26.54	8.69	20.41	6.37	16.86	20.00	13.71	8.69
Total n-3	22.65	21.30	19.05	20.82	44.18	41.66	49.51	20.55	20.45	19.05	14.00	31.40	24.31	33.70	38.38	24.12	21.78
n-6/n-3	0.44	0.33	0.25	0.58	0.09	0.06	0.30	0.09	0.05	0.10	0.68	0.01	0.38	0.14	0.07	0.00	0.00
Others	7.67	3.66	23.72	8.91	0	4.64	0.01	6.44	7.57	0.12	4.6	1.56	0	5.88	0	14.78	13.08
Total PUFA	40.31	32.08	38.10	41.83	48.18	48.61	64.12	28.88	29.04	39.51	28.10	33.36	33.55	44.24	41.11	38.38	39.60

FA: fatty acids; SFA: saturated fatty acids; MUFA: monounsaturated fatty acids; PUFA: polyunsaturated fatty acids.

Table S13. Fatty acids profile (mg/100g) of Fish / Shellfish / Crustaceans sub-category.

mg/100g																	
	Seabream fillet		Sea bass		Cod		Sole	Salmon		Tuna	Farmed Eel	Anchovy	Mussel	Clams	Frozen shelled prawns	Lobster	Octopus
	Caught	Farmed	Caught	Farmed	Fresh	Frozen		Fresh	Smoked								
C12:0	0.00	0.00	0.00	0.00	0.00	0.52	1.21	0.00	0.00	0.00	0.00	0.00	0.00	0.00	0.00	0.00	0.00
C14:0	43.74	243.95	37.12	327.18	0.05	11.99	29.56	510.92	186.92	268.47	899.56	123.52	67.53	40.71	43.30	0.00	34.27
C16:0	711.94	1399.40	308.94	875.03	52.13	96.16	116.67	1995.90	747.29	2345.39	2829.52	892.12	392.07	210.39	138.48	342.49	239.88
C18:0	186.12	371.03	86.58	182.42	2.60	24.04	29.56	413.30	157.33	268.47	541.37	114.75	243.46	53.38	43.30	117.23	85.67
Others	22.03	42.36	0.00	0.00	7.79	0.00	0.00	1.04	10.51	16.82	0.00	17.55	0.00	39.05	8.57	108.19	0.00
Total SFA	963.84	2056.74	432.64	1384.63	62.57	132.71	177.00	2921.16	1102.06	2899.14	4270.44	1147.94	703.06	343.52	233.65	567.91	359.82
C14:1n-6	0.00	0.00	0.00	0.00	0.00	0.00	0.00	0.00	0.00	0.00	112.85	0.00	0.00	0.00	0.00	0.00	0.00
C16:1n-7	142.39	487.89	74.12	547.85	52.13	11.99	78.63	639.69	235.99	190.66	1421.30	88.20	216.36	80.79	34.58	0.00	17.13
C18:1n-9	679.06	1823.75	259.61	1076.87	20.85	87.70	127.82	2419.59	904.62	822.22	4690.78	150.07	216.36	89.51	164.41	288.39	68.54
Others	197.31	636.16	3.63	423.10	0.00	32.71	59.12	1464.21	550.64	19.63	1207.04	114.97	419.17	13.92	34.66	171.33	85.67
Total MUFA	1018.75	2947.80	370.86	2047.82	72.98	132.40	265.57	4523.49	1691.25	1306.58	7431.98	353.25	851.89	184.22	233.65	459.72	171.34
C18:2n-6	120.36	445.53	12.33	356.02	0.00	0.00	0.12	147.46	19.86	129.68	1032.04	8.77	67.53	13.40	17.29	0.00	0.09
C20:2n-6	0.00	0.00	0.00	0.00	0.00	0.00	0.00	0.00	0.00	0.00	0.00	0.00	0.00	0.00	0.00	0.00	0.00
C20:3n-6	0.00	0.00	0.00	0.00	0.00	0.00	0.00	0.00	0.00	0.00	0.00	0.00	0.00	0.00	0.00	0.00	0.00
C20:4n-6	208.16	74.50	49.46	356.02	10.44	11.99	177.00	48.81	19.86	0.70	521.74	0.22	148.84	35.00	3.97	0.16	0.00

Total n-6	328.51	520.03	61.79	712.03	10.44	11.99	177.12	196.27	39.72	130.38	1553.78	9.00	216.36	48.39	21.26	0.16	0.09
C18:3n-3	10.85	53.32	12.33	19.42	0.00	0.00	68.81	88.27	29.60	77.81	50.70	8.77	53.97	12.15	8.65	0.00	0.00
C20:3n-3	0.00	0.00	0.00	0.00	0.00	0.00	0.00	0.00	0.00	0.00	0.00	0.00	0.00	0.00	0.00	0.00	0.00
C20:5n-3	317.66	495.20	111.24	461.35	31.28	36.03	216.38	875.41	324.39	692.54	817.78	238.50	365.20	162.73	138.48	162.45	154.21
C21:5n-3	0.00	0.00	0.00	0.00	0.00	0.00	0.00	0.00	0.00	0.00	0.00	0.00	0.00	0.00	0.00	0.00	0.00
C22:5n-3	0.00	0.00	0.00	0.00	0.00	0.00	0.00	0.00	0.00	0.00	0.00	0.00	0.00	0.00	0.00	0.00	0.00
C22:6n-3	416.31	1007.19	1.30	744.40	83.41	180.27	314.63	1170.33	442.38	1860.33	1421.30	459.22	148.84	175.08	155.77	225.42	75.20
Total n-3	744.83	1555.70	247.28	1225.16	114.70	216.31	599.83	2134.01	796.36	1335.32	2289.78	706.49	568.01	349.96	302.89	396.58	188.48
n-6/n-3	0.44	0.33	0.25	0.58	0.09	0.06	0.30	0.09	0.05	0.10	0.68	0.01	0.38	0.14	0.07	0.00	0.00
Total PUFA	1325.56	2343.05	494.56	2461.51	125.11	252.40	776.83	2999.04	1130.87	2769.47	4595.92	750.59	784.13	459.41	320.18	631.05	342.69
Total	3308.15	7347.59	1298.06	5893.96	260.66	517.51	1219.4	10443.69	3924.18	6975.19	16298.34	2251.78	2339.08	987.15	787.48	1658.68	873.85

FA: fatty acids; SFA: saturated fatty acids; MUFA: monounsaturated fatty acids; PUFA: polyunsaturated fatty acids.

Table S14. Fatty acids profile (% of total FA) of Cheeses sub-category

% of total FA									
	**Aged cheese**	**Mixed caciotta**	**Cow ricotta**	**Sheep ricotta**	**Creamy blue cheese**	**Yogurt from whole milk**	**Whole milk**	**Semi-skimmed milk**	**Butter**
C12:0	3.70	3.99	3.89	6.24	2.69	3.94	3.29	3.51	3.18
C14:0	12.10	11.91	13.26	13.80	9.25	11.83	11.09	11.93	11.07
C16:0	28.61	28.75	34.80	26.94	30.25	30.24	27.48	31.58	27.79
C18:0	10.93	10.38	10.57	7.37	13.35	11.18	11.65	14.74	12.52
Others	10.64	10.89	5.49	15.22	8.69	10.86	9.51	0.70	10.43
Total SFA	65.98	65.92	68.01	69.57	64.23	68.05	63.02	62.46	64.99
C14:1n-6	0.00	0.00	1.10	0.38	0.00	0.00	2.09	0.00	1.52
C16:1n-7	2.28	0.00	1.89	1.51	3.37	0.33	2.99	2.81	2.53
C18:1n-9	29.04	26.90	26.63	20.70	28.77	27.28	27.78	30.18	27.55
Others	0.03	0.14	-2.10	1.04	0.10	0.00	0.00	-0.01	0.00
Total MUFA	31.35	27.04	27.52	23.63	32.24	27.61	32.86	32.98	31.60
C18:2n-6	1.35	4.40	2.49	3.02	2.05	2.63	2.09	3.51	2.09
C20:2n-6	0.00	0.00	0.00	0.00	0.00	0.00	0.00	0.00	0.00
C20:3n-6	0.00	0.00	0.00	0.00	0.00	0.00	0.00	0.00	0.00
C20:4n-6	0.00	0.00		0.00	0.00	0.00	0.00	0.00	0.00
Total n-6	1.35	4.40	2.49	3.02	2.05	2.63	2.09	3.51	2.18
C18:3n-3	1.49	2.87	1.40	3.31	1.28	1.64	1.49	2.11	1.57

C20:3n-3	0.00	0.00	0.00	0.00	0.00	0.00	0.00	0.00	0.00
C20:5n-3	0.00	0.00	0.00	0.00	0.00	0.00	0.00	0.00	0.00
C21:5n-3	0.00	0.00	0.00	0.00	0.00	0.00	0.00	0.00	0.00
C22:5n-3	0.00	0.00	0.00	0.00	0.00	0.00	0.00	0.00	0.00
C22:6n-3	0.00	0.00	0.00	0.00	0.00	0.00	0.00	0.00	0.00
Total n-3	1.49	2.87	1.40	3.31	1.28	1.64	1.49	2.11	1.57
n-6/n-3	0.23	1.53	1.78	0.91	1.60	1.60	1.40	1.66	1.39
Total PUFA	2.81	7.27	3.89	6.43	3.33	2.81	3.58	5.61	3.66

FA: fatty acids; SFA: saturated fatty acids; MUFA: monounsaturated fatty acids; PUFA: polyunsaturated fatty acids.

Table S15. Fatty acids profile (mg/100g) of Cheeses sub-category

mg/100g									
	**Aged cheese 1**	**Mixed caciotta**	**Cow ricotta**	**Sheep ricotta**	**Creamy blue cheese**	**Yogurt from whole milk**	**Whole milk**	**Semi-skimmed milk**	**Butter**
C12:0	950.96	1070.38	366.93	620.99	630.85	132.97	102.50	45.56	2295.08
C14:0	3109.90	3195.05	1250.76	1373.35	2169.28	399.26	345.49	154.86	7989.47
C16:0	7353.24	7712.65	3282.54	2681.02	7094.13	1020.59	856.10	409.93	20056.68
C18:0	2809.19	2784.60	997.02	733.45	3130.80	377.32	362.94	191.33	9035.97
Others	2734.65	2921.42	517.85	1514.67	2037.95	366.52	296.27	9.09	7527.57
Total SFA	16957.94	17684.11	6415.10	6923.48	15063.00	2296.66	1963.29	810.77	46904.76
C14:1n-6	0.00	0.00	103.76	37.82	0.00	0.00	65.11	0.00	1097.02
C16:1n-7	586.00	0.00	178.28	150.27	790.32	11.14	93.15	36.48	1825.96
C18:1n-9	7463.76	7216.36	2511.90	2060.03	6747.04	920.69	865.44	391.76	19883.46
Others	7.71	37.56	-198.08	103.50	23.45	0.00	0.00	-0.13	0.00
Total MUFA	8057.46	7253.92	2595.85	2351.61	7560.82	931.83	1023.70	428.10	22806.44
C18:2n-6	89.96	1180.37	234.87	300.54	480.76	88.76	65.11	45.56	1508.40
C20:2n-6	0.00	0.00	0.00	0.00	0.00	0.00	0.00	0.00	0.00
C20:3n-6	0.00	0.00	0.00	0.00	0.00	0.00	0.00	0.00	0.00
C20:4n-6	0.00	0.00	0.00	0.00	0.00	0.00	0.00	0.00	0.00
Total n-6	89.96	1180.37	234.87	300.54	480.76	88.76	65.11	45.56	1573.36

C18:3n-3	382.95	769.92	132.06	329.40	300.18	55.35	46.42	27.39	1133.10
C20:3n-3	0.00	0.00	0.00	0.00	0.00	0.00	0.00	0.00	0.00
C20:5n-3	0.00	0.00	0.00	0.00	0.00	0.00	0.00	0.00	0.00
C21:5n-3	0.00	0.00	0.00	0.00	0.00	0.00	0.00	0.00	0.00
C22:5n-3	0.00	0.00	0.00	0.00	0.00	0.00	0.00	0.00	0.00
C22:6n-3	0.00	0.00	0.00	0.00	0.00	0.00	0.00	0.00	0.00
Total n-3	382.95	769.92	132.06	329.40	300.18	55.35	46.42	27.39	1133.10
n-6/n-3	0.23	1.53	1.78	0.91	1.60	1.60	43.70	21.59	1.39
Total PUFA	722.22	1950.30	366.93	639.90	780.94	94.84	111.53	72.82	2641.51
Total	25737.62	26888.33	9377.88	9914.99	23404.76	3323.33	3098.52	1311.69	72352.71

FA: fatty acids; SFA: saturated fatty acids; MUFA: monounsaturated fatty acids; PUFA: polyunsaturated fatty acids.

Table S16. Fatty acids profile (% of total FA) of Eggs sub-category.

% of total FA				
	**Laying hen**			
	**Chicken egg**	**Yolk conventional**	**Yolk Bio-plus**	**Duck egg**
C12:0	0.00	0.00	0.61	0.00
C14:0	0.43	0.18	1.92	0.38
C16:0	27.30	25.49	26.82	22.92
C18:0	17.39	33.13	30.26	4.81
Others	0.43	3.10	4.12	0.00
Total SFA	45.55	61.90	63.73	28.11
C14:1n-6	0.00	0.00	0.20	0.00
C16:1n-7	3.30	0.31	0.50	3.36
C18:1n-9	33.76	32.44	32.47	46.45
Others	0.01	0.56	0.00	0.00
Total MUFA	37.07	33.31	33.50	49.81
C18:2n-6	15.23	3.10	3.24	19.56
C20:2n-6	0.00	0.00	0.00	0.00
C20:3n-6	0.00	0.00	0.00	0.00
C20:4n-6	2.30	0.96	0.00	2.44
Total n-6	17.53	4.06	3.24	22.00
C18:3n-3	0.57	0.18	0.29	0.76
C20:3n-3	0.00	0.00	0.00	0.00
C20:5n-3	0.00	0.00	0.00	0.00
C21:5n-3	0.00	0.00	0.00	0.00
C22:5n-3	0.00	0.00	0.00	0.00
C22:6n-3	0.00	0.00	0.00	0.00
Total n-3	0.57	0.18	0.29	0.76
n-6/n-3	30.75	22.56	11.17	28.95
Total PUFA	18.01	4.24	3.73	22.77

FA: fatty acids; SFA: saturated fatty acids; MUFA: monounsaturated fatty acids; PUFA: polyunsaturated fatty acids.

Table S17. Fatty acids profile (mg/100g) of Eggs sub-category

mg/100g				
	Laying hen			
	**Chicken egg**	**Yolk conventional**	**Yolk Bio-plus**	**Duck egg**
C12:0	0.00	0.00	0.00	0.00
C14:0	32.37	0.00	95.00	50.64
C16:0	2055.35	5300.00	4700.00	3054.50
C18:0	1309.25	2610.00	1820.00	641.02
Others	32.37	901.37	195.00	0.00
Total SFA	3429.35	8210.00	6810.00	3746.16
C14:1n-6	0.00	0.00	0.00	0.00
C16:1n-7	248.45	0.00	725.00	447.78
C18:1n-9	2541.71	10060.00	11440.00	6190.29
Others	0.75	162.83	0.00	0.00
Total MUFA	2790.91	11200.00	12165.00	6638.07
C18:2n-6	1146.63	2600.00	1880.00	2606.72
C20:2n-6	0.00	0.00	0.00	0.00
C20:3n-6	0.00	0.00	0.00	0.00
C20:4n-6	173.16	5880.00	110.00	325.17
Total n-6	1319.79	8480.00	1990.00	2931.89
C18:3n-3	42.91	130.00	334.00	101.28
C20:3n-3	0.00	0.00	31.00	0.00
C20:5n-3	0.00	29.00	15.00	0.00
C21:5n-3	0.00	0.00	0.00	0.00
C22:5n-3	0.00	15.00	15.00	0.00
C22:6n-3	0.00	87.00	501.00	0.00
Total n-3	42.91	261.00	896.00	101.28
n-6/n-3	30.75	32.49	2.22	28.95
Total PUFA	1355.93	2600.00	3330.00	3034.51
Total	7576.19	22010	22305	13418.74

FA: fatty acids; SFA: saturated fatty acids; MUFA: monounsaturated fatty acids; PUFA: polyunsaturated fatty acids.
